# Supplementary material for: Laser-induced phase separation of silicon carbide
Source: Nat Commun. 2016 Nov 30;7:13562. doi: 10.1038/ncomms13562 (PMC5141366; doi:10.1038/ncomms13562)
Supplement: Supplementary Information — Supplementary Figures 1-10, Supplementary Table 1 and Supplementary Notes 1-3 [file ncomms13562-s1.pdf]

# SUPPLEMENTARY FIGURES

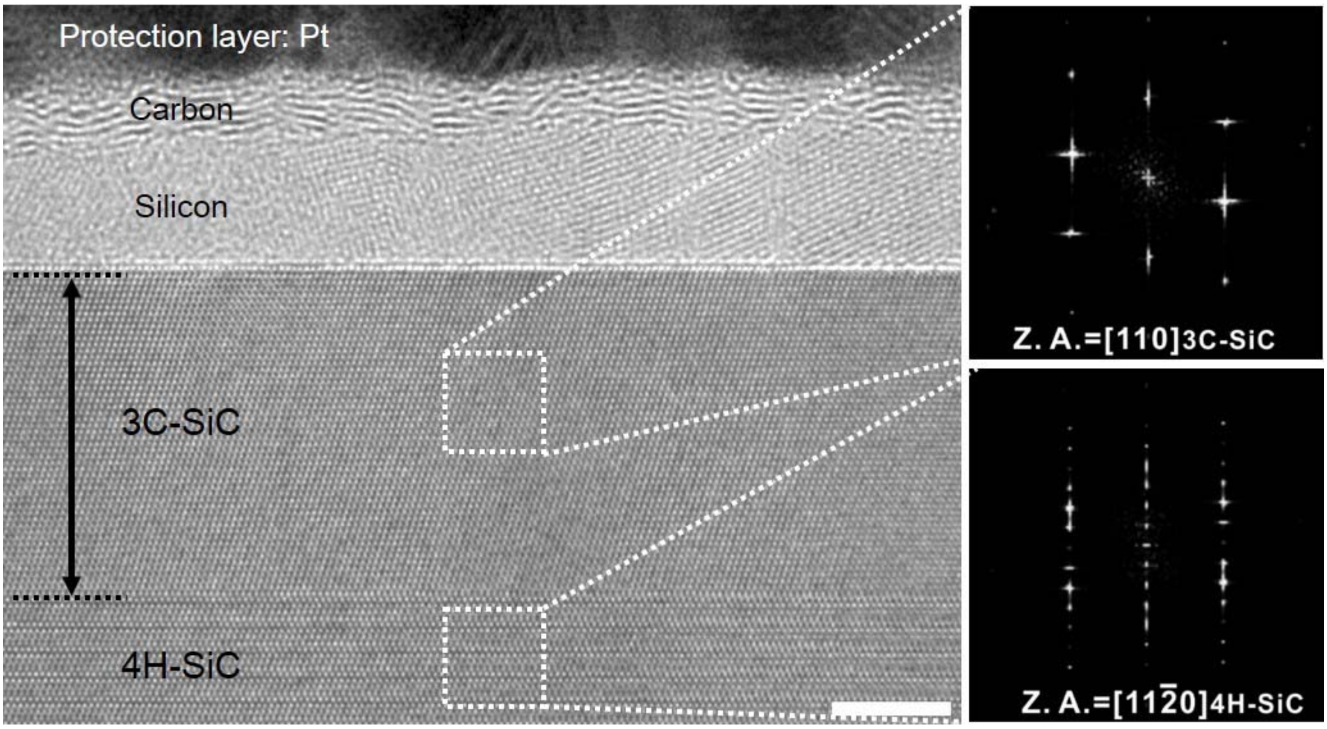


**Supplementary Figure 1** | HRTEM image of a single-pulse irradiated 4H-SiC surface. FFT patterns clearly show a (111)-oriented 3C-SiC layer and an (0001)-oriented 4H-SiC substrate. Scale bar = 5 nm.

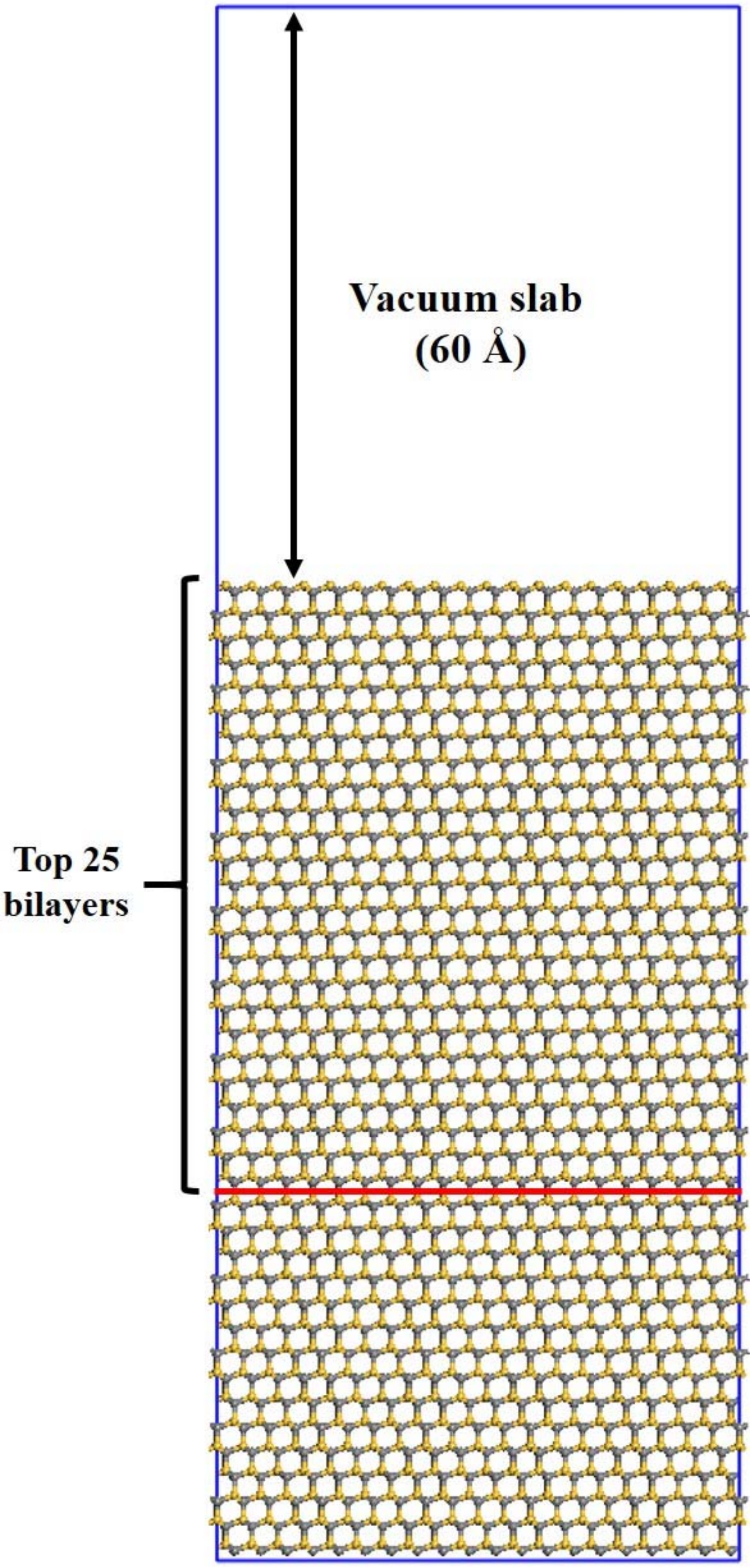


**Supplementary Figure 2 |** Side view of the Si-face of a (111)-oriented 3C-SiC substrate. Gray and yellow spheres represent C and Si atoms, respectively. The periodic boundary condition box is shown as a blue solid line.

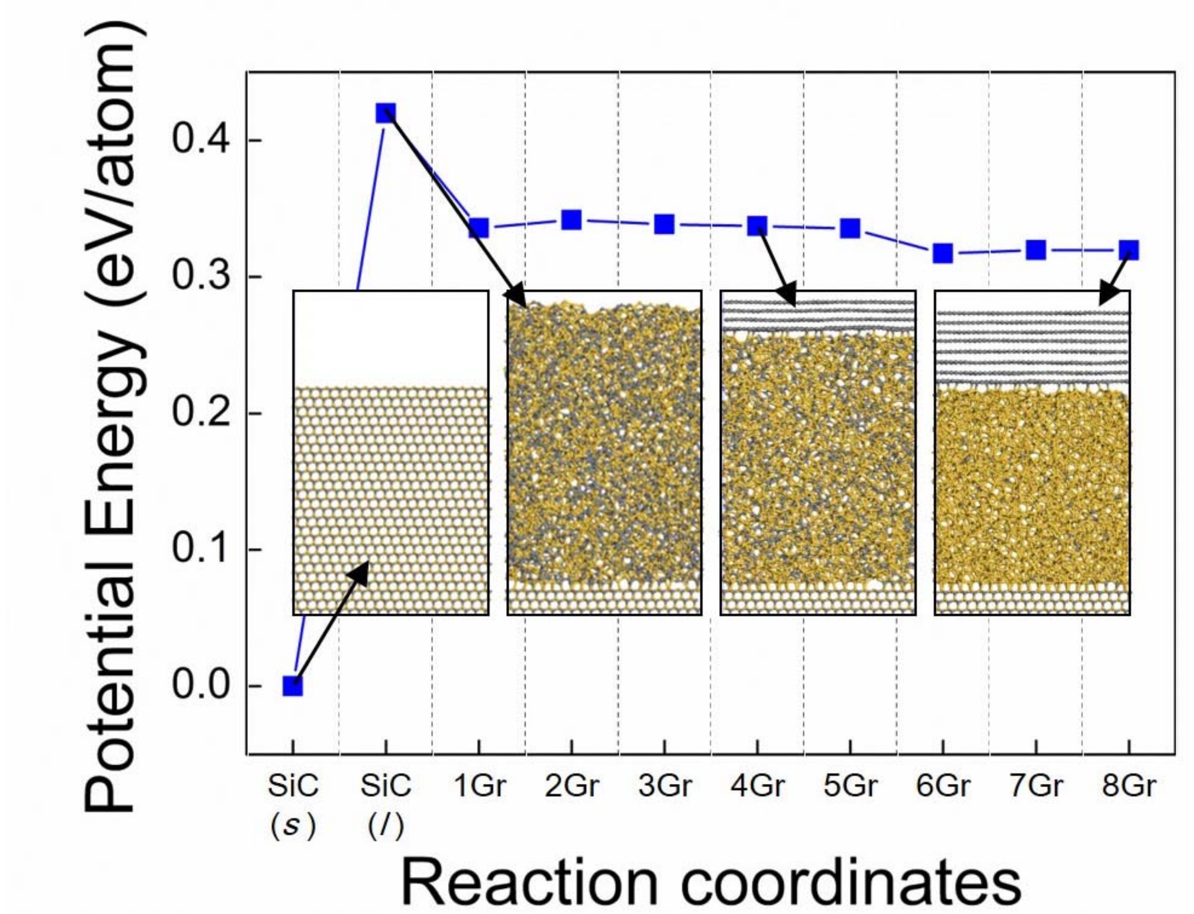


**Supplementary Figure 3** | Potential energy plot for the formation process of multilayer graphene from the SiC system. Each step is optimized by performing MD simulation and the insets show the side view of each step taken from the MD simulations. All simulation details are described in the "Molecular dynamics simulation" section. After SiC (*s*) is transformed into SiC (*l*) by the high-energy laser source corresponding to thermal treatment up to approximately 3500 K, it reaches a metastable state with surface graphene layers. During this process, C atoms are extracted from the SiC (*l*) surface and are converted into a multilayer graphene structure by decreasing the overall potential energy of SiC (*l*) layer atoms. In other words, the potential energy reduction induces a thermodynamic driving force to generate graphitic C layers from the SiC (*l*) surface and to consequently reach the metastable state. Gr indicates one layer of carbon (graphene).

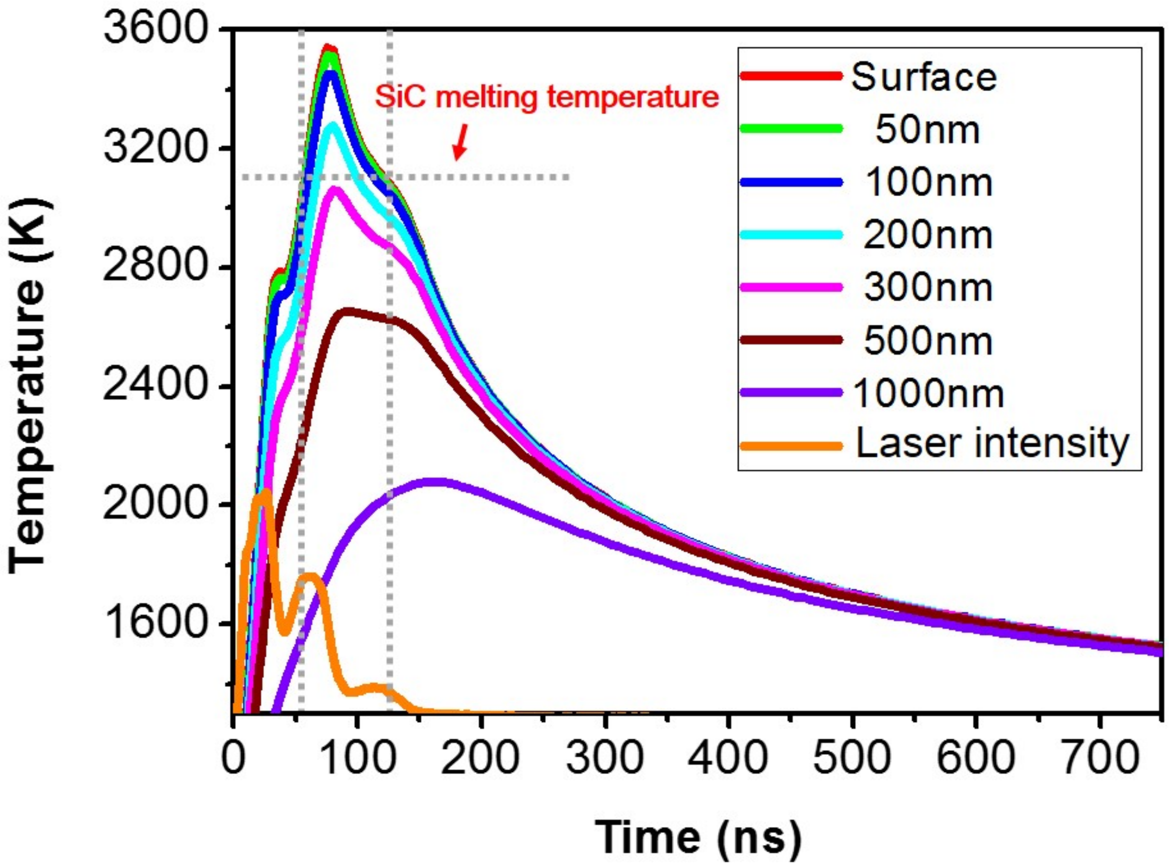


**Supplementary Figure 4** | Temperature distributions for a 4H-SiC surface irradiated with a single-pulse by one-dimensional numerical simulation. Simulated temperatures were investigated in various positions of surface, 50, 100, 200, 300, 500, and 1000 nm. There are very similar temperatures from the top surface to a depth of 50 nm due to the high thermal conductivity of single-crystal SiC. The surface temperature was increased to 2784 K by the first hump of laser beam, which is below the melting point of SiC. The second hump of laser beam leads to an accumulation of thermal energy and the surface temperature increases to 3541 K at 75 ns. This simulated temperature indicates that laser fluence supplies enough thermal energy to melt the SiC surface.

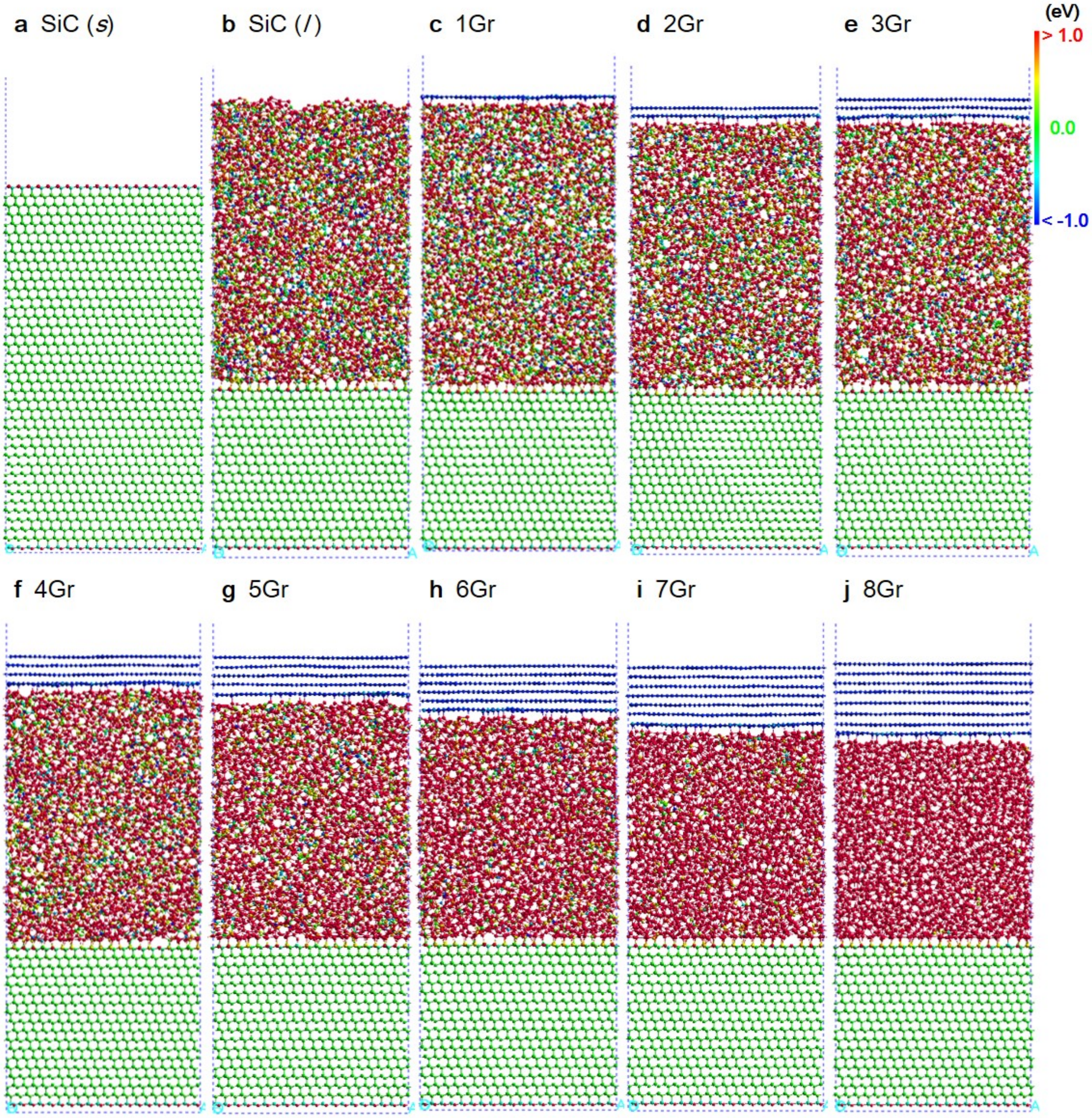


**Supplementary Figure 5** | Atomic potential energy spectra of the simulation systems optimized by performing MD simulations. The color spectrum bar in the right-top indicates the relative potential energy referenced to the energies of bulk atoms. Red and blue colors describe a high potential energy regime and a low potential energy regime, respectively. After SiC (*s*) is transformed into SiC (*l*) by a high-energy laser source, the surface atoms arrange themselves into graphene which reduces the surface potential energy. Eventually, the surface potential energy reduction by graphitic C layer formation induces a thermodynamic driving force to reach the metastable state even though the potential energy of the residual atoms in $SiC_{1-x}$ (*l*) below the surface graphene layers remains high. Gr indicates one layer of carbon (graphene).

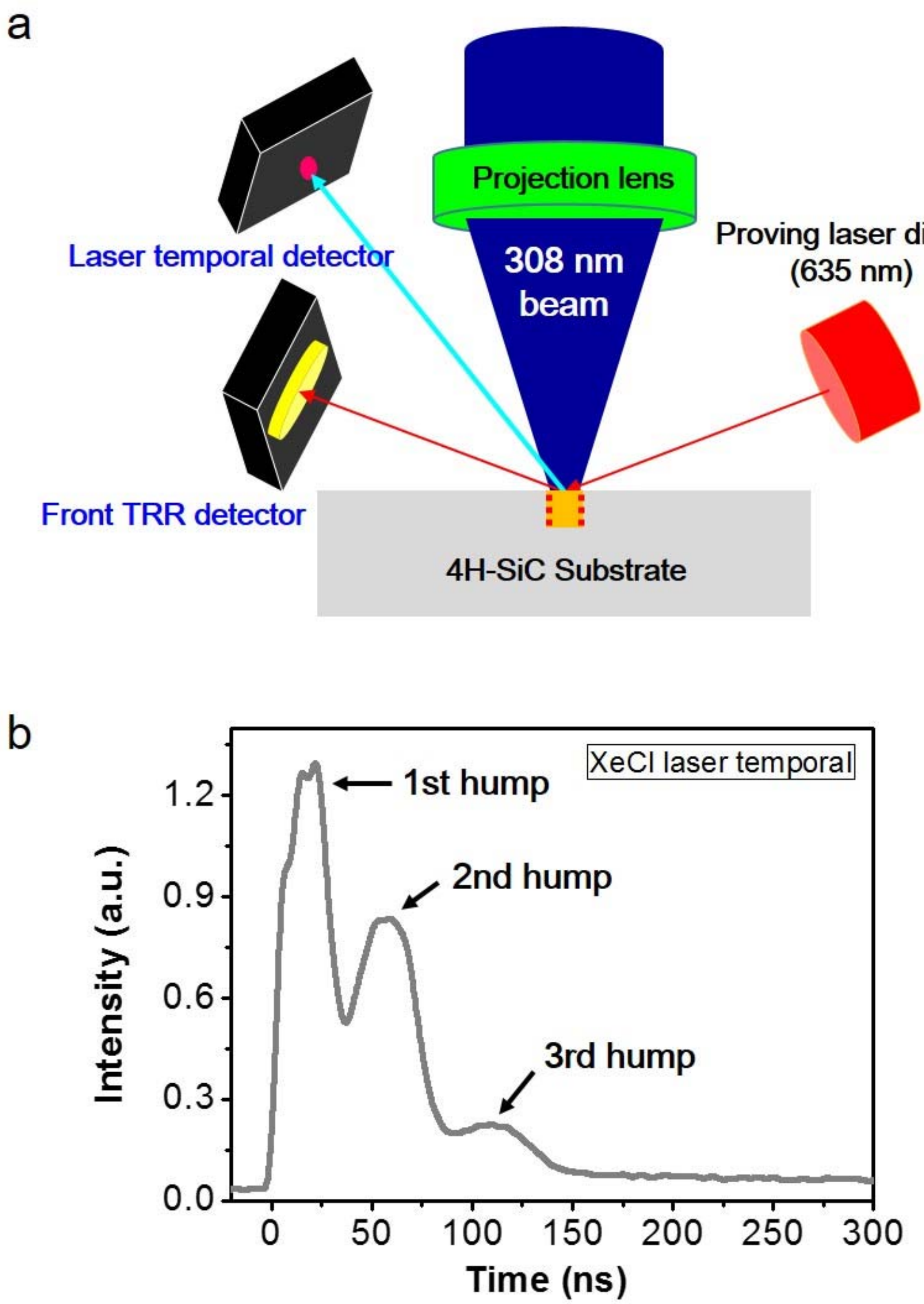


**Supplementary Figure 6** | (**a**) Schematic of the measurement system of time-resolved reflectance analysis (**b**) Laser intensity as a function of time (laser temporal) of 308 nm XeCl excimer laser (Coherent, LPX model). Single-pulse was measured by using photo detector and oscilloscope.

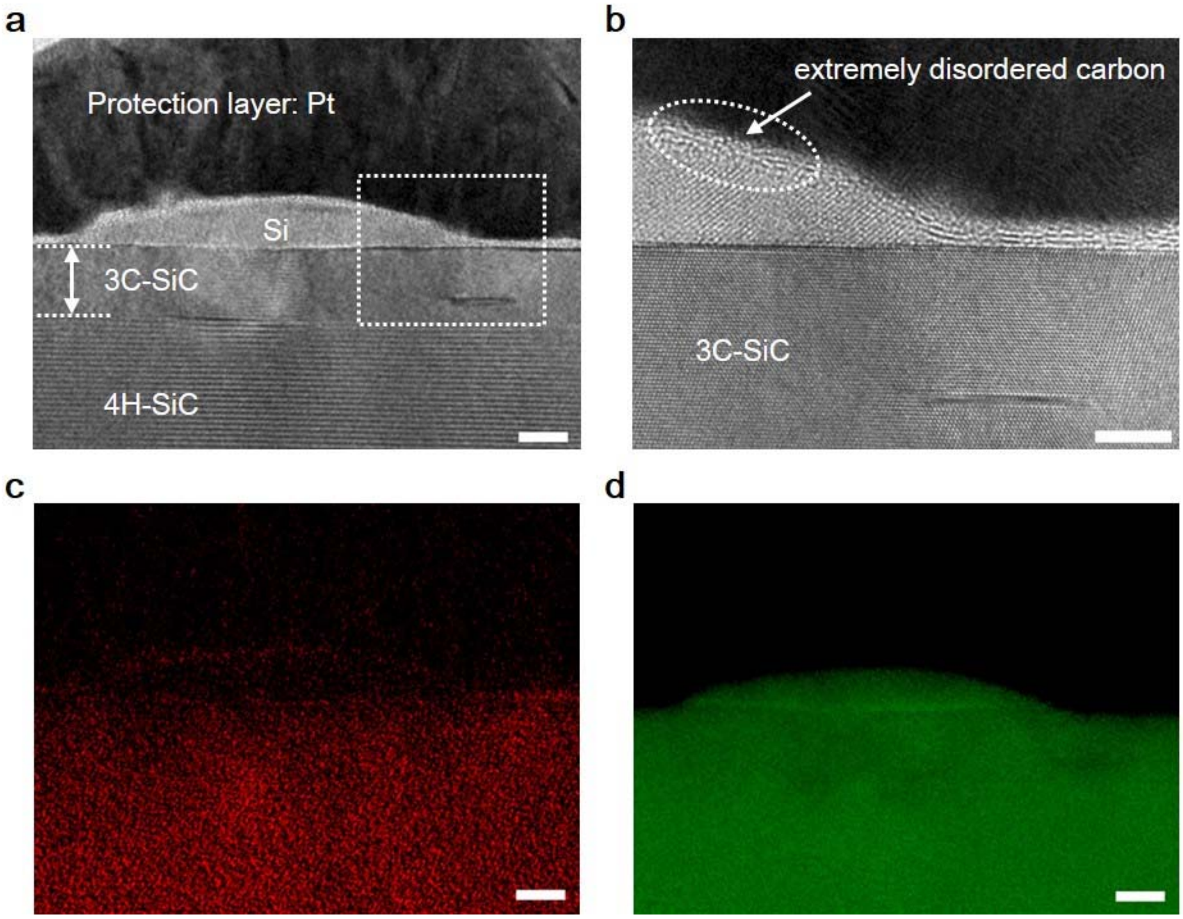


**Supplementary Figure 7** | (**a**) HRTEM image of a non-uniform area on 4H-SiC surface after two irradiation pulses. Scale bar = 10 nm. (**b**) Magnified image of white-dotted rectangle area in **a**. Scale bar = 5 nm. (**c**, **d**) EFTEM mapping images of C K- and Si L-edge. Scale bars = 10 nm.

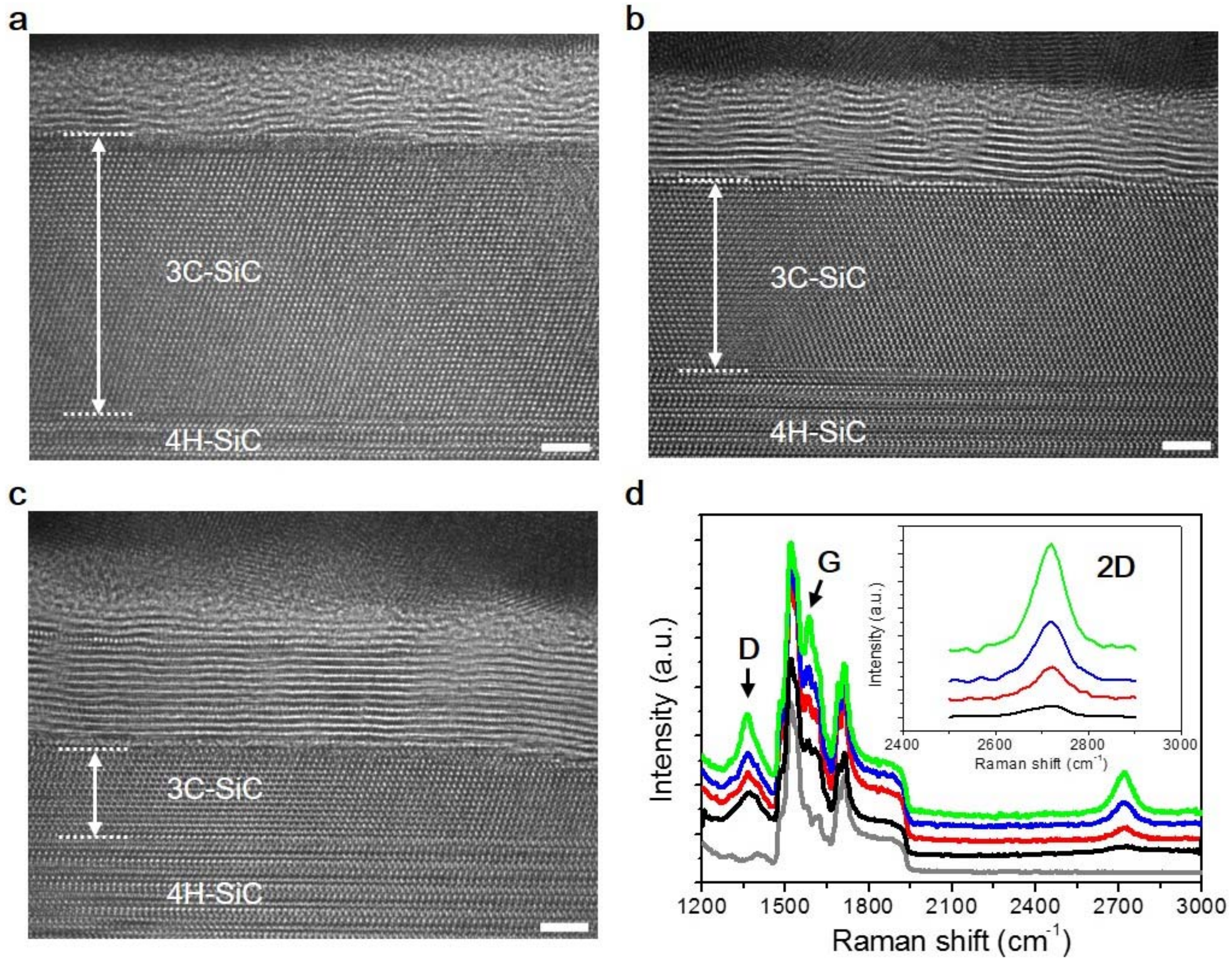


**Supplementary Figure 8** | HRTEM images of 4H-SiC surface after 30 (**a**), 100 (**b**), and 300 (**c**) irradiation pulses. Scale bars = 2 nm. Double-sided arrows indicate 3C-SiC layers. (**d**) Raman spectra of laser-induced multilayer graphene on 4H-SiC surfaces after irradiation of 30 (black line), 100 (red line), 200 (blue line), and 300 pulses (green line). Three representative peaks, including the defect-induced D peak, in-plain vibrational G peak, and two phonon scattered 2D peak are clearly observed at 1364, 1583, and 2720 $cm^{-1}$, respectively. The inset shows magnification of 2D peak. The gray line is the Raman spectrum for an original 4H-SiC substrate as a reference.

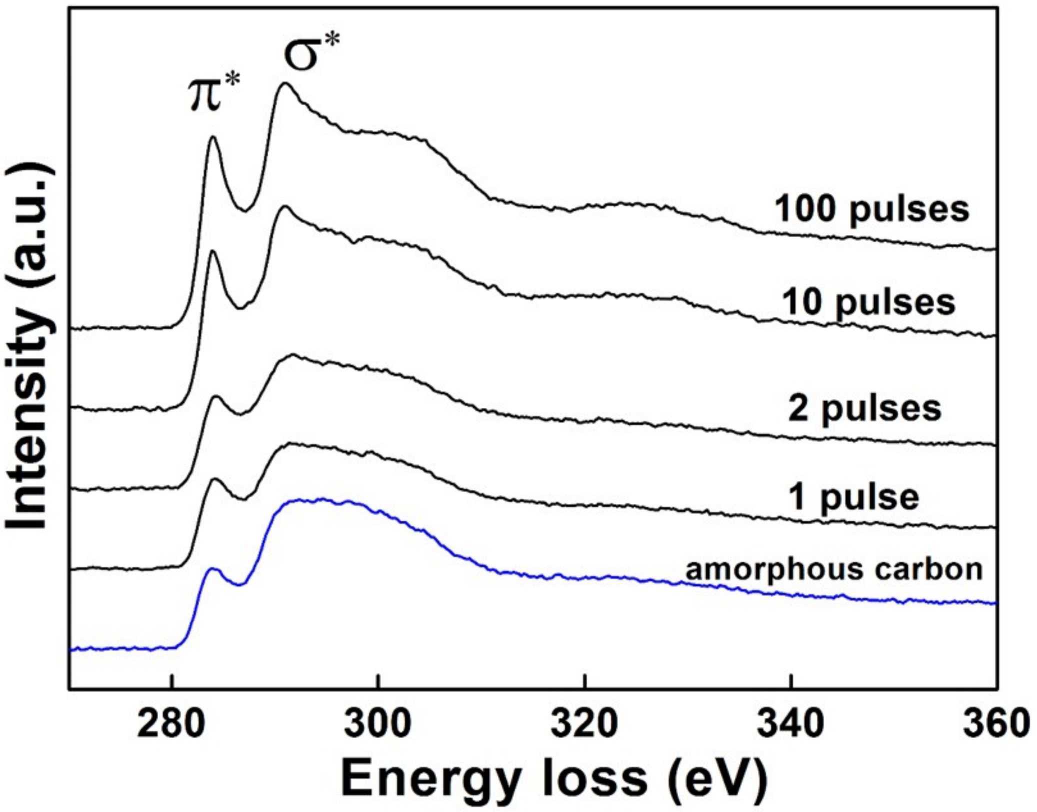


**Supplementary Figure 9 |** EELS spectra of C layers formed by multi-pulse irradiation. Both data of ten and one hundred irradiations show a highly graphitic ordered structure which is comparable to CVD-grown multilayer graphene.

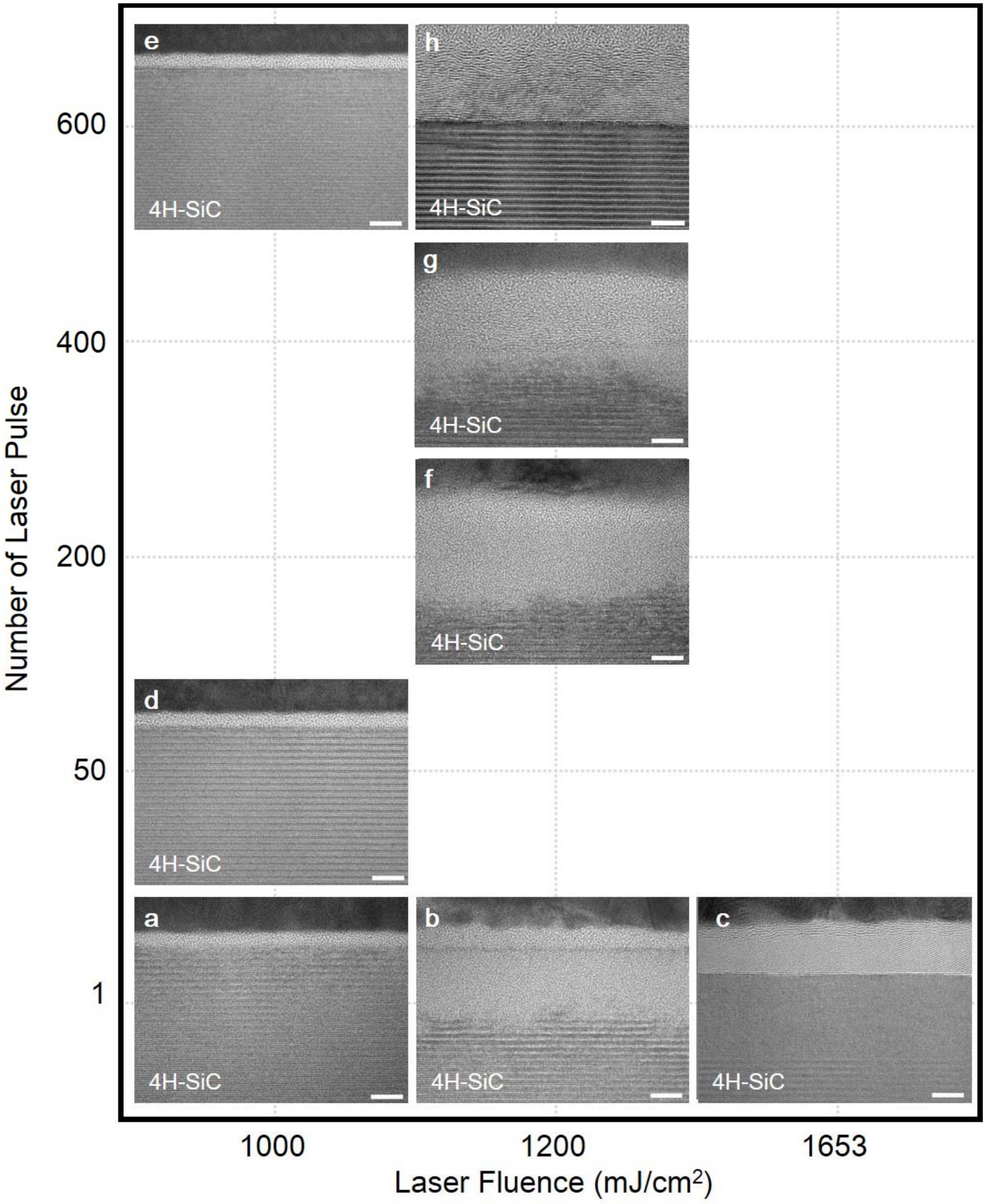


**Supplementary Figure 10** | Investigation of laser-SiC interaction as a function of laser fluence and the number of irradiation pulses. HRTEM images of 4H-SiC surface after single-pulse irradiation with laser

fluence of 1000 (**a**), 1200 (**b**), and 1653 mJ $cm^{-2}$ (**c**). HRTEM images of 4H-SiC surface after 50 (**d**) and 600 (**e**) irradiation pulses with laser fluence of 1000 mJ $cm^{-2}$. HRTEM images of 4H-SiC surface after 200 (**f**), 400 (**g**), and 600 (**h**) irradiation pulses with laser fluence of 1200 mJ $cm^{-2}$. Scale bars = 5 nm. Our study indicates two different mechanism with melt-mediated phase separation or amorphous phase transition for the formation of C layer on single-crystal SiC surface, compared to the conventional thermal decomposition[16-18] and 248 nm KrF laser irradiation[19]. We estimate that 308 nm XeCl laser has a higher possibility to melt SiC surface due to its longer pulse duration (which includes the second hump at laser intensity, Supplementary Fig. 6b) and a higher absorption depth at 308 nm wavelength, compared to effects of 248 nm KrF laser[19].

## SUPPLEMENTARY TABLE

| Model | #Atoms | Lattice parameters (Å) | | | Volume (Å$^3$) |
|---|---|---|---|---|---|
| | | *a* | *b* | *c* | |
| Graphite | 8 | 4.26 | 2.46 | 6.67 | 8.75 |
| Cubic Si | 8 | 5.41 | 5.41 | 5.41 | 19.75 |

**Supplementary Table 1** | The number of atoms, lattice parameters, and volumes for each unit cell after geometry optimization steps.

# SUPPLEMENTARY NOTES

## Supplementary Note 1

**Time-resolved reflectance analysis of single-pulse irradiation:** We divided the time-resolved reflectance (TRR) signal into six different regions corresponding to observed signal changes to analyze the phase separation and surface reconstructions of single-crystal SiC. The first region indicates the lowest reflectance of 4H-SiC surface before laser irradiation. The intensity of reflectance at 37 ns (the second region) was slightly increased, which corresponds to the hot solid state (before melting) by irradiation of the first hump of laser beam. The third region was determined from 50 to 180 ns including a peculiar shape at 75 ns. The TRR signal was remarkably increased from 50 to 75 ns by supplying the second hump of laser pulse, indicating melting of 4H-SiC. The unusual high intensity at 75 ns is estimated to include the reflectance signals from the 4H-SiC surface and the laser intensity. Phase separation was considered to be produced before complete solidification of the liquid SiC layer in the third region. The thicknesses of the melt-mediated phase separation region (~7.5 nm) and the transition layer (3C-SiC, ~15 nm) were very thin, compared to the heat diffusion length (~1 um) estimated from the heat transfer equation, $\sqrt{2D\tau}$[22,23,27]. Therefore, thickness up to several hundreds of nanometers could be melted and recrystallized to 4H-SiC, whereas only a thin layer (~15 nm) under the separated layer was transformed to 3C-SiC which is a stable phase in non-equilibrium conditions. A long flattened region was observed from 180 to 450 ns (fourth region), which demonstrates the existence of a liquid phase. The TRR signal of the fifth region, showing the final signal change, between 450 and 480 ns shows solidification indicating the coexistence of liquid and solid phases. This solidification process only involves Si due to its low melting temperature (~1700 K), compared to that of SiC (~3100 K). The TRR signal of the sixth region after 480 ns is very stable, which means complete cooling of surface.

## Supplementary Note 2

**TRR analysis of the second irradiation pulse:** We analyzed melt-mediated surface reconstructions with six different regions corresponding to changes of the TRR signal. The reflectance of the first region indicates a graphitic C surface before the second irradiation pulse. The first hump of laser beam caused a remarkable increase of reflectivity at 37 ns, unlike the small increase in the single-pulse irradiation at the same time (Fig. 1g). This is related to the initial surface structure of graphitic C/poly-Si/3C-SiC on 4H-SiC. The poly-Si layer was melted and evaporated as soon as irradiation of the first hump of laser beam. The third region corresponding to a decrease in the TRR signal was determined to be from 50 to 150 ns. Similar to the results for the single-pulse irradiation, an unusually high intensity was observed at 75 ns by supplying the second hump of laser beam. Both 3C-SiC and 4H-SiC were molten at this time. A flattened region indicating a liquid phase between 150 and 290 ns (the fourth region) is much shorter than for the single-pulse irradiation, as shown in Fig. 1g. On the other hand, this flattened region indicates liquid SiC because a separated Si layer was not detected on the surface, as shown in Fig. 4b. The TRR signal of the fifth region between 290 and 320 ns presents solidification indicating coexistence of liquid and solid phases of SiC. After 320 ns (the sixth region), the TRR signal was stable, which means complete cooling of the surface. The intensity of the TRR signal during the six regions was very similar to that of the first region, because both regions indicate the existence of the same C material on the surface before and after the second irradiation pulse, which is in agreement with the HRTEM analyses.

**Supplementary Note 3**

**TRR analysis of multi-pulse irradiation:** The TRR signal for the 4H-SiC surface irradiated with third pulse shows a very similar spectrum shape to that of the second irradiation pulse. This means that there was no further generation of a Si layer by phase separation. Additional TRR data from 20 to 100th pulse show a similar spectrum shape including the flattened period. Therefore, the thickness increase of the C layer is believed to occur by the addition of C atoms from the interface between the bottom C layer and the liquid SiC surface through laser-induced melt-mediated decomposition of SiC.
